# The stochastic world of emerging viruses

**DOI:** 10.1093/pnasnexus/pgac185

**Published:** 2022-09-12

**Authors:** Laurent Gavotte, Roger Frutos

**Affiliations:** Espace-Dev, Université de Montpellier, Montpellier 34070, France; Cirad, Intertryp, Montpellier 34070, France

## Abstract

The acquisition of new hosts is a fundamental mechanism by which parasitic organisms expand their host range and perpetuate themselves on an evolutionary scale. Among pathogens, viruses, due to their speed of evolution, are particularly efficient in producing new emergence events. However, even though these phenomena are particularly important to the human species and therefore specifically studied, the processes of virus emergence in a new host species are very complex and difficult to comprehend in their entirety. In order to provide a structured framework for understanding emergence in a species (including humans), a comprehensive qualitative model is an indispensable cornerstone. This model explicitly describes all the stages necessary for a virus circulating in the wild to come to the crossing of the epidemic threshold. We have therefore developed a complete descriptive model explaining all the steps necessary for a virus circulating in host populations to emerge in a new species. This description of the parameters presiding over the emergence of a new virus allows us to understand their nature and importance in the emergence process.

Significance Statement The emergence of a new virus in a host population is a complex process. The characteristics of emerging epidemics are well described after the epidemic threshold is crossed, as their dynamics become deterministic and are similar to those of classical epidemics. However, the steps and parameters that influence them before this threshold are stochastic in nature. This work therefore proposes a complete descriptive modeling of the different stages leading to a new emergence. This step is an important milestone toward a better understanding of these processes.

## Introduction

The emergence of a transmissible disease is, by definition, the establishment of an epidemic dynamic within a host species by a pathogen that has not previously infected it. By nature, such events are rare and result from complex processes whose explanatory parameters are mostly unknown. One of the main problems we face with emerging communicable diseases is that we only perceive these diseases when they have already emerged and have caused many visible and quantifiable cases. Indeed, from a medical standpoint (human or veterinary), a disease emergence is only formalized when a clinical picture, thus a disease, is associated with a novel pathogen. However, this clinical phase and an ongoing epidemic represent only the last part of the long and complex process of disease emergence. The dynamic of declared epidemics is now well known and the mathematical modeling is precise and well mastered (1–4). However, it is the only well-characterized stage. There is still a dark zone between the time when a new pathogen infects the first individual of the new host species (the primary case) and the moment when the disease is identified and characterized in the first patient to be recognized as affected by this novel disease (the index case). Studies have been developed to try to assess an epidemic potential of known emerging viruses (5). There are attempts to model segments of the emergence process in different ways, such as recent work modeling the infection dynamics of SARS-CoV-2, based on available genomic data (6). The process of infection of the primary case, however, is the subject of ongoing debates. Currently, two models exist to explain this dark zone, the spillover model (7) and the circulation model (8). However, these models themselves are only attempts to describe the key steps necessary for an emerging disease to appear, and not the description of the mechanism per se. The present work aims to focus exclusively on the mechanisms involved in the phase preceding an epidemic caused by an emerging virus. We will therefore only address in this work the period from the circulation in the wild and the infection of an individual of a new host species (primary case) to the crossing of the epidemic threshold within this new species.

Among the diversity of microorganisms with a potential as emerging disease causative agents, viruses, and more specifically RNA viruses, are the most problematic candidates. Most emerging or re-emerging human viral diseases are caused by RNA viruses. The WHO in its “Prioritizing diseases for research and development in emergency contexts list”, lists no less than 10 diseases, 9 of whthemeing caused by RNA viruses, and the remaining one being the theoretical disease “X” whose etiological agent is not known yet (4). The periodic emergence of Ebola virus in human populations is a perfect example of these conceptual weaknesses. Indeed, Ebola disease is easy to detect due to its dramatic pathogenic effect and the associated high death rate. Furthermore, nearly each Ebola virus epidemic is the result of a unique emergence process, which could allow to build a model of disease emergence and validate it, owing to the numerous field data acquired. The current model assumes that the Ebola virus infects populations of fruit bats, and from there the infection reaches humans either directly by bat/human contact or indirectly through a bat/intermediate animal/human process (9). However, this theoretical process is not supported by evidence. Anti-Ebola antibodies have indeed been detected punctually in fruit bats (10–12), as well as traces of genomic material of the virus (12). However, no active virus has ever been isolated. The active circulation of the virus in these bat species and thus their status as source of human infections cannot be certified. Wild animals other than bats were found dead at the time and place of human Ebola epidemics (13). However, it was never clarified whether these animals were the origin of the human infection or instead have been contaminated by infected humans. Finally, the origin of the latest outbreak in Guinea (February to June 2021) was shown to be related to a virus already circulating for several years in the human population (14). No spillover from any animal species occurred (14). Such limitations, while the Ebola virus is particularly tracked and studied suggest that the currently accepted concepts of virus emergence are at best unsatisfactory.

It is necessary to decipher the process of virus emergence and describe in detail the various steps. We describe in this article an in-depth and detailed analysis of each step of the process and provide a model describing all these steps.

## The concept of filters

The model presented in this article builds on the “parasitic filters” developed in the 1980s by Louis Euzet and Claude Combes to describe the sorting processes restricting the host range of parasite, but was updated since then (15–18). These filters correspond to two successive and complementary entities: the encounter filter and the compatibility filter. The encounter filter defines the probability for a pathogenic organism to encounter a potential host. It includes a spatiotemporal component (overlay of ecological niches or at least simultaneous presence of host and pathogen) and an ethological component (behavior allowing transmission of the pathogen), e.g. the blood-feeding behavior of mosquitoes allowing the transfer of many pathogens. The compatibility filter defines the probability for a pathogen to achieve an infection, i.e. at least a transmission to the next host. It includes a molecular component (molecular dialogue between host and parasite including recognition, manipulation etc.), a physiological component (providing space and resources for the pathogen), and an immunological component (evading the host's innate or induced immune defense mechanisms).

## Description of the model

**Fig. 1.**
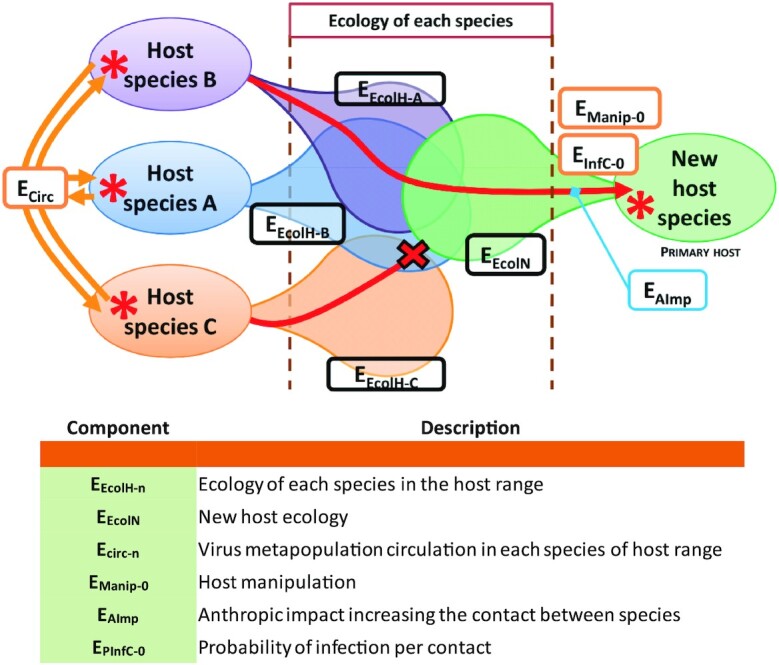
Initial environmental step of virus emergence process. Red lines illustrate the possible pathways for virus emergence at this stage.

**Fig. 2.**
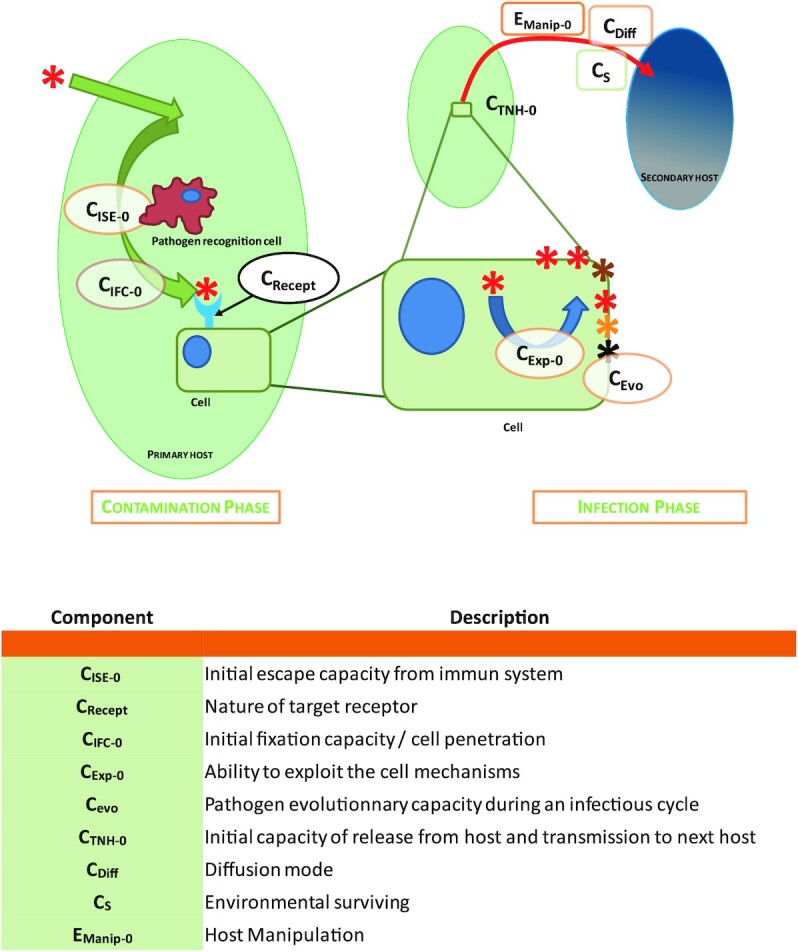
Primary infection step of virus emergence process. Red lines illustrate the possible pathways for virus emergence at this stage.

**Fig. 3.**
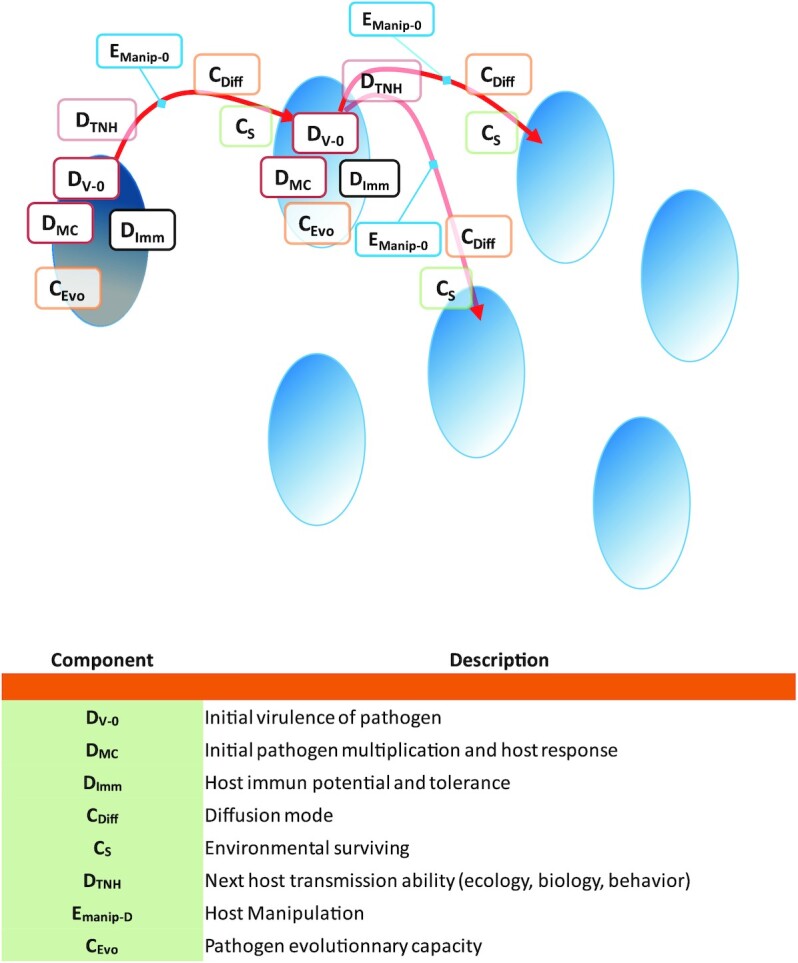
Low-noise new host dynamic step of virus emergence process. Red lines illustrate the possible pathways for virus emergence at this stage.

**Fig. 4.**
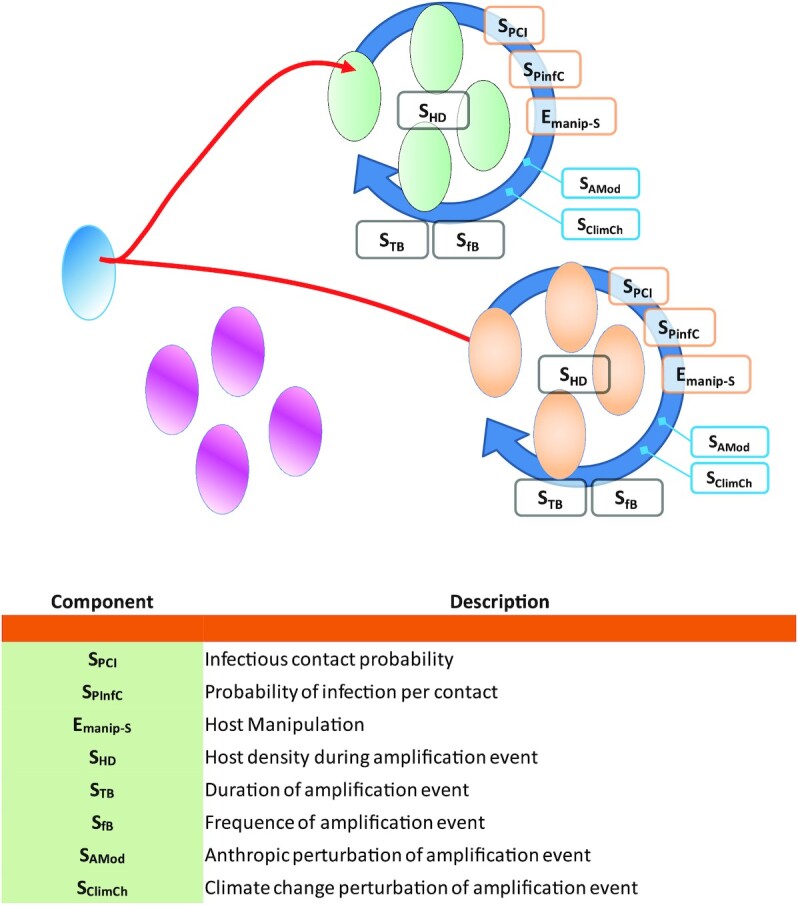
Amplification loop step of virus emergence process. Red lines illustrate the possible pathways for virus emergence at this stage.

The emergence of a virus in a new host species can be divided into three obligatory successive phases: (i) an initial environmental phase (also referred to as the sylvatic phase) (Fig. 1), which is characterized by the natural life cycle and dissemination dynamic of the virus, (ii) a primary infection phase (Fig. 2), which starts when the first individual comes into contact with the virus, and (iii) the intra-host transmission (Figs. 3 and 4), which will determine the epidemic or even pandemic dimension of the disease.

During the environmental phase, a virus metapopulation circulates between different hosts and adapt differentially by intra-host selection to the different species. Each host species has the potential to harbor a genetically differentiated population of the virus metapopulation depending on the ecological contact between the host species and their respective host genetic differences. This host range is largely defined by the target receptor recognized by the virus to enter and infect a target cell. However, viruses from any host species remain capable of triggering an infection in another species within its host range. From a “pathogen point of view,” the concept of species barrier does not cover a reality as only the presence of a receptor recognizable by the virus proteins determines the possibility of infection (13). The virus must thus be physically in contact with an individual of the novel species. This first encounter filter is modulated by different parameters:

The level of circulation of the virus metapopulation in each species within its host range—*E_circ_*. A high circulation increases the probability of encounter and transmission. A high number of host species in the host range induces a high network of circulating viruses. This parameter is very difficult to estimate due to the very limited knowledge on the dispersion of viruses in the environment when it is not simply absent. Such viruses circulating among various host species were already identified. For example, Rodrigues et al., in 2017 (19) considers that, among the known virosphere, 73.3% of viruses are associated with only one or two host species; 3.5% with three or four species and 22.5% with more than four species. Modeling was also attempted (20, 21) but the real network of transmission often remains cryptic. Systematic screening using metagenomic methods on the basis of representative fauna (or environmental) samples could be used to estimate this parameter.

The ecology of each species in the host range—*E_E__colH__-n_*. This parameter covers the habitat, population density, and behavior of the potential donor host species—*E_E__colH__-A_, E_EcolH__-B_* etc and intensity of contact with individuals of the recipient host species according to its own ecology—*E_E__col__N_*. However, complex ecological niche descriptions exist for many host species, such as bats (22).

The probability of encounter can be directly and deeply modified in some cases if the virus is able to manipulate and modify the behavior of its host—*E_M__anip__-0_-* (e.g. rabies virus) (20).

The human-induced changes in the environment—*E_AImp_-* will alter the way in which the different species in the ecosystem can encounter, either directly (e.g. modification of habitats, displacement of species outside their range or pollution) or indirectly (e.g. climate change modification of the ecological niches, behaviors, such as long-distance migrations, or the overall health condition of individuals) (23–25). One of the best documented cases is the dispersal across continents of vector species via human-made transportation and trade, which results in an increase in the range of the diseases they may transmit (26–28).

The last parameter to consider for the passage of this first filter and establishment of a primary infection is the transmission capacity of the virus—*E_P__Inf__C__-0_*. This capacity is the result of the mode of transmission, thus the location of the viral replication sites in the initial host, the density of viable viral particles produced, and the survival competence of the virus in the environment at the time of the transmission (29). If this parameter cannot be estimated for an unknown virus, it is possible to perform laboratory experiments (e.g. infection of cultured cell lines) in the case of already isolated viruses.

Once the virus particles have entered an individual of the novel potential host species, a primary infection can take place. But for this to be successfully achieved, the virus must pass through a compatibility filter, which, if not passed, will stop any potential viral emergence. This filter is controlled by different parameters (Fig. 2):

The virus must escape the host immune system—*C_ISE-0_*–composed at this stage of innate and nonspecific responses but also potentially adaptive responses in the case of cross-reactions with other pathogens having previously infected the host. A recently reported example is the immune protection against the emerging Mayaro virus following Chikungunya infection in humans and mice (30, 31). This may represent a possible way to study this parameter.

The host cell surface receptor—*C_Recept_*-, recognized by the external proteins of the virus, thus allowing to initiate the internalization process and initiate infection is particularly important. Indeed, each virus recognizes only a very limited range of host receptors, often only one molecule and sometimes a co-receptor, and therefore its recognition “window” is extremely limited (32). If the proper receptor or epitope is not present on the cells in contact with the pathogen (not encoded by the host genome or not expressed in the cells present in the zone of presence of the viral particles), there will simply be no infection. The nature of the target receptors is therefore fundamental, so a receptor that is widely distributed in vertebrates (and therefore most likely encoded by a housekeeping gene under high neutralizing selection pressure) will allow the virus to potentially infect many host species. This is the case for example for the ACE2 receptor used by sarbecoviruses, which are theoretically able to infect numerous vertebrates (33, 34). On the other hand, a more restricted receptor or widely divergent in its amino acid sequence between taxa will limit the virus more strongly. Mutations inhibiting an initially efficient receptor, or modification of epitopes, will lead to the same result. For example, among poxviruses, some have a wide host spectrum (such as cowpox, which can effectively infect rodents, felids, canines, bovines, or humans), while others can only infect a few or even a single species (as is the case for the smallpox virus) (35).

The initial ability of the virus to bind to the target receptor of its new host—*C_IFC-0_*—is influenced by the diversity of the receptor of the new species compared to the initial host species and the tolerance of the virus to this variability. In vitro or in silico studies could be used to evaluate these cross-binding capacities.

The virus ability to use correctly the cellular machinery—*C_Exp-0_*—will condition the production of new viruses. It is expected that protein or nucleic acid chain synthesis process are similar at the large evolutive scale, whereas other processes could significantly differ with evolutive distance between species. In vitro or in silico studies could be used to evaluate these abilities.

The virus will then disseminate in the primary infected host by infecting new cells and new cell types. At this stage, the virus ability to evolve at each infection cycle—*C_E__vo_*—will allow it to adapt more rapidly to its new host. The quasi-species evolutive mode displayed by RNA viruses is the source of a particularly important potential for adaptation (36).

The virus ability to release virions from its host to infect a new individual host within the newly infected species—*C_TNH-0_*—will determine whether the primary infection could initiate a network of contamination in the newly infected population or remain restricted to an isolated infection. This capacity will be impacted by the organs where the virus replicates, its mode of dissemination—*C_Diff_*—and its ability to survive in the environment—*Cs*.

This probability of encounter can be directly altered in some cases if the virus is able to manipulate and modify the behavior of its host—*E_Manip-0_*.

To continue the emergence process, the virus will pass new encounter and compatibility filters at each infection of a new individual. The dynamics of the infection will remain low-key, however, and it will take specific conditions for the infection to cross the outbreak (epidemic) threshold and trigger an emerging disease. Below this threshold, the pathogen may disappear (and probably disappears most of the time) due to stochastic events.

A discrete intranovel host species transmission allows the virus to maintain the infection. This also allows time for adaptive selection mechanisms to optimize the virus infection capacities to its new host species. The genome has been selected until then in the previously infected species and will now evolve to adapt more specifically to this novel host species. This low-key diffusion is conditioned by a new encounter filter defined by different factors:

The initial replication capacity of the pathogen (in its primary infection form)—*D_V__-__0_*—and its evolution will largely affect its transmission for two reasons: (a) too low a replication capacity will not allow the production of sufficient viral particles for an efficient transmission and survival (the effective population might be too small) and (b) too high a replication capacity will decrease the mobility of the infected host, increase its probability of dying or decrease its social interactions (avoidance behavior from healthy individuals), and will finally result in a decrease of the virus transmission (37).

The virus virulence is controlled by the pathogen multiplication capacity—*D_MC_*—(and damage caused to the host) as well as the immune response of the host—*D_Imm_*—, which will modulate the virulence of the virus (possibly to its advantage within the limits of what was explained for *D_V-0_*), increasing or decreasing the achieved virulence according to various mechanisms: (a) neutralization of infectious viruses and infected cells decreases virulence, (b) immune tolerance to limit the inflammatory response decreases virulence, and (c) a poorly controlled immune response will increase tissue damage and thus final virulence (38).

The mode of transmission of the virus—*C_Diff_*—in combination with its ability to survive in the environment—*C_S_*—will determine the capacity of the virus to infect another host. For example, the main transmission routes of the hepatitis E virus vary depending on the genotype. Genotypes 1 and 2 are preferentially transmitted to humans through contaminated water, while genotypes 3 and 4 are mainly transmitted through the consumption of meat from infected animals (39).

The capacity of transmission to the next host**—***D_TNH_*—will depend on several biological, ecological, and ethological parameters of the new host species. Finally, the behavior of the host can also be modified and manipulated by the virus—*E_manip-D_*.

The virus evolutionary capacities—*C_Evo_*—will, with each new cycle, potentially select mutations that gradually optimize the viral genome to the characteristics of its new host.

However, this virus population remains at too low a frequency in the host population to be safe from random elimination. At this stage, there is no identifiable outbreak, perhaps some sporadic cases, in the human population but only chains of contamination that can be interrupted. To become epidemic, the level of infection must cross the outbreak (epidemic) threshold (2) and this can only happen during specific events in which conditions will suddenly favor the diffusion of the infection in the host population: the amplification loops or superspreading events. Amplification loops result in a fast increase in the local virus demography. An amplification loop remains stochastic in nature and it is only once the outbreak threshold is crossed that the dynamics become linear and deterministic and therefore the classical indicators (R0, incidence, etc.) can be calculated. These amplification loops are of social nature and characterized by a significant increase in the density of interaction between individuals of the host species. Although their efficiency depends in part on the transmission capacity of the virus, their existence and dynamics depend only on the biology and behavior of the host, although the virus can significantly modify it if the host is manipulated. When referring to the human species, these amplification loops are societal events driven by societal rules. These amplification loops are controlled by different parameters of an encounter filter:

Specific elements of virus and host interactions, namely the probability of an infecting contact between two individuals—*S_PCI_*—, depends essentially on the level of virus circulation in the host population, but also on the probability of successful infection in case of contact—S_PInfC_. The virus can also modify this last parameter if it is able to manipulate its host—E_manip-D_—, which can be modulated over time by the evolution of the virus.

Concerning the amplification loops themselves, they are characterized by three main parameters: (a) the host population density—*S_HD_*—during the event, (b) the duration of the event—*S_TB_*, and (c) the frequency of the event—*S_fB_*. The amplification loops are fractal by nature. Each event will impact the following ones, leading to an increase in the prevalence of the infection in the populations concerned. The superspreading events, often referred to as clusters, detectable in the deterministic phases of different epidemics are the epidemiologic counterpart of these amplification loops, they respond to the same constraints (40–42).

The different parameters of these loops can also be modified (increased or decreased) by various anthropogenic disturbances—*S_AMod_*—(e.g. reducing the habitat surface area will increase the probability of encounters) but also by climate change—*S_ClimCh_*—, which will disturb the distribution of species but also their behavior, e.g. their circadian movements or migrations.

Once a virus over runs the outbreak threshold owing to the occurrence of amplification loops (several consecutive events could be necessary), the epidemic is declared, the disease emergence is achieved, and a cluster of clinical cases will be generated. The dynamics of the disease now follow classical epidemiological deterministic models.

## Discussion

The emergence of new pathogens, i.e. still unknown pathogens at the time of its emergence such as SARS-CoV-2, whether they are viruses as in this model or any other pathogen, is a process that is particularly difficult to apprehend and understand for two main reasons:

- The dynamic of pre-epidemic emerging pathogenesis is, by definition, extremely complex to assess. Indeed, at any given moment, there are countless primary cases of a variety of pathogens in all possible species. A recent survey on games and wild animals in China showed the presence of 102 viruses, out of which 21 were considered as posing a potential risk to humans (43). However, the vast majority of these primary cases disappear without leaving any trace in the epidemiological history of these species. Serological data can provide valuable but limited information. However, we cannot yet estimate, which pathogen, within this multitude of potentialities, has the capacity to become an emerging one and whether this process of emergence will be successful.
- The dual chaotic/deterministic nature of the disease emergence phenomenon makes it difficult to understand and to develop tools and models. If the deterministic phase is now well determined and considerable scientific progress has been made, the chaotic phase remains completely unexplored and largely misunderstood.

The difference in nature, stochastic/chaotic vs. deterministic, of the mechanisms involved in the emergence processes of viral diseases implies a mathematical modeling approach beyond the qualitative model described in this work. The deterministic postoutbreak threshold phase has been well studied, described, and modeled for several decades and the success of predictions based on classical epidemiological models demonstrates its relevance. This is not the case for the stages preceding the crossing of the outbreak threshold and the detection of an epidemic in a population. This black box clearly cannot be modeled in a classical way for two main reasons.

First, the complexity of the parameters involved would need to be broken down and each subparameter evaluated and modeled. The ecology of each species in the host range is summarized by *E_EcolH-n_* parameters, but it covers many aspects of the biology and ecology of each species, including ecological niche (which is already a complex parameter), behavior (whose modeling is, in essence, complicated), etc.

Second, while some parameters follow deterministic laws, such as the match between the virus binding proteins and the cell receptor present in the new host species, chance plays a central role in the achievement of many events. For example, the actual encounter of an individual of a host species, effectively infected by the virus, with an individual of the new host species is of course influenced by many parameters (shared ecological niche, behavior, prevalence, population density, etc.), but the realization of the event is fundamentally random.

The chaotic nature of disease emergence does not allow the application of classical deterministic models. This fact is perfectly illustrated by the very existence of an epidemic threshold, described by Hartfield and Alizon (2). The behavior of the stochastic dynamics of complex systems is very difficult to model, especially since biologists are not used to manipulating such concepts. It seems, however, that there can exist many stable states during the process of emergence and that the continuation of the processes can be done only under certain conditions that lead, after a chaotic transition, to another stable state. For example, the initial state of presence of the virus in its initial host population is a stable state (in the sense that it follows deterministic laws of classical epidemiology, even if they can be complex to understand and model). The perturbation consisting in the acquisition of a new host species by the virus (i.e. the emergence process itself) leads to a complex chaotic dynamic characterized by a succession of contamination and transmission conditioned by the parameters deciphered in the model developed here. The final result can only consist of two alternative stable states, i.e. (i) a loss of infection in the new species or (ii) the crossing of the epidemic threshold. Such a mechanism can be similar to the bifurcation phenomenon widely studied, described, and modeled in physics. A bifurcation can be defined as a change of state of a nonlinear (or complex) system when the value of a so-called control parameter reaches a critical value. The macroscopic nature of the system changes, although its microscopic nature remains the same. In addition to the complete process of disease emergence, it is possible to identify bifurcations at different key steps of the process: the infection of the primary host of the new host species, the successful infection of the primary case, and each of the amplification loops.

In order to model and therefore study disease emergence processes, it will be necessary to develop a new approach including methods to model these stochastic dynamics that resemble the deterministic chaos known in physics. Part of the answer will undoubtedly be to adapt concepts and tools developed for physical phenomena that have similarities with the mechanisms involved in pathogen emergence.The future of emergence risk studies will rely in particular on spatial approaches and on the elaboration of increasingly complex risk maps integrating numerous parameters and generated by state-of-the-art computer tools such as machine learning techniques (44). The development of a probabilistic epidemiology integrating this (r)evolution in our understanding of these mechanisms is an achievable goal in the coming decades.

## Authors' Contributions

L.G. and R.F. designed research, performed research, and wrote the paper.

## Data Availability

There are no data underlying this work.
